# Hippocampal neuronal cyclooxygenase-2 downstream signaling imbalance in a rat model of chronic aluminium gluconate administration

**DOI:** 10.1186/s12993-015-0054-z

**Published:** 2015-02-18

**Authors:** Hong Wang, Mengliang Ye, Lijuan Yu, Jianfeng Wang, Yuanxin Guo, Wenjuan Lei, Junqing Yang

**Affiliations:** Department of Pharmacology, Chongqing Medical University, Key Laboratory of Biochemistry and Molecular Pharmacology, Chongqing, 400016 China; Department of Biostatistics, School of Public Health, Chongqing Medical University, Chongqing, Chongqing, 400016 China

**Keywords:** Aluminium gluconate, Neurotoxicity, Cyclooxygenase, Prostaglandins, Prostaglandin synthases, Prostaglandin receptors

## Abstract

**Background:**

Acute and chronic brain damages including neurodegenerative diseases are a group of neuroinflammation-associated diseases characterized by cognitive function defect and progressive neuron loss. The pathophysiological procession of brain damages involves the overexpression of cyclooxygenase (COX)-2. Owing to the limited benefit to chronic brain damage and the late adverse effect of COX-2 inhibitors, the COX downstream signaling pathway has become a focus in neurological research. In order to explore the mechanism of aluminum neurotoxicity and the importance of COX2 downstream signaling pathways to chronic brain damage, the present study was designed to simultaneously observe the prostaglandin (PG) contents, and the expressions of PG synthases and PG receptors of hippocampus in a rat model induced by chronic administration of aluminium gluconate.

**Methods:**

A rat model of chronic brain damage was established by chronic intragastric administration of aluminium gluconate (Al^3+^ 200 mg/kg per day, 5d a week for 20 weeks). PG contents, the expressions of PG synthases, and the expressions of PG receptors in rats were measured by ELISA, RT-PCR and Western blotting, respectively.

**Results:**

Chronic aluminium gluconate administration resulted in hippocampal neuron injury and learning and memory disorders in rats. Aluminium gluconate administration also resulted in increased levels of PGE2, PGD2, TXA2, PGI2, and PGF2α in rat hippocampus. The DP1, EP2, IP, mPGES-1, EP4, PGIS and TXAS mRNA expressions, and the DP1, EP2 and IP protein expressions significantly increased in the Al-treated hippocampus, while the EP3 and FP mRNA and protein expressions and the TP mRNA expression decreased.

**Conclusions:**

The PGS/PGs/PG receptors signaling pathway in chronic aluminium gluconate-overloaded rat hippocampus is disturbed, which may be involved in the mechanism of aluminium neurotoxicity.

## Background

Aluminum (Al), which accounts for about 8% of the earth's crust composition, is the most ubiquitous metal in water, soil and air and is extensively used in modern daily life. Therefore, we unavoidably take in Al through various routes, including the use of pharmaceuticals (e.g. antacids, buffered analgesics, antiulceratives, and antidiarrheal drugs). The daily Al intake by humans through the gastrointestinal tract is up to 126–5000 mg/day [1], corresponding to 12–500 mg/kg daily by rats converted via the body surface area index between humans and rats [2]. However, the physiological action of Al on humans is unclear. Since the first report of Al toxicity to humans at early 1970s, it has been identified that Al overload could cause severe brain damage and neurodegeneration [3]. In particular, Al neurotoxicity could be involved in the pathogenesis of Alzheimer’s disease (AD) and Al was detected in senile plaques and neurofibrillary tangles in the brain tissues from AD patients [1,3]. The mechanism of Al neurotoxicity is still unknown. As reported, the Al-induced neuronal injury is closely related to neuroinflammatory response and oxidative stress [4-6].

Cyclooxygenase (COX) can catalyze arachidonic acid (AA) to bioactive prostaglandin (PG)H2. PGH2 can be further catalyzed by specific PG synthases (PGS) to PGs, including PGE2, PGD2, thromboxane A2 (TXA2), prostacyclin (PGI2), and PGF2α. COX has two isoenzymes: the constitutive COX-1 and the inducible COX-2. COX-1 is constitutively expressed in many tissues, including the brain and especially the microglia. COX-2 is also highly and basally expressed in many tissues, including brain neuronal cells. COX-2 is mainly present in the cell bodies and dendritic regions of neurons of hippocampus, cerebral cortex, and amygdale [7]. COX-2 expression in neurons can be induced by excitotoxic insults,transient global cerebral ischemia and oxidative stress as well as inflammatory mediators [8,9]. Hippocampal and frontal cortex neuronal COX-2 mRNA and protein levels will significantly increase in the brains of AD patients [5,10].

We previously found that chronic intragastric (i.g.) administration of aluminium gluconate (Al^3+^ 200 mg/kg per day) caused significant increase of hippocampal metal ion levels (Al, Fe, Mn, Cu and Zn), and induced learning and memory function disorders in rats [11]. We also find that the aluminium gluconate administration-induced chronic brain damage in rats can be remarkably prevented by meloxicam, a COX-2 inhibitor [12]. These findings suggest that the over-expression of COX-2 may play an important role in the occurrence of neuron damage and neurodegeneration, and the inhibitors of COX-2 may prevent the acute and chronic brain damages induced by aluminium gluconate.

However, the potential beneficial effect is limited to some non-steroidal antiinflammatory drugs (NSAIDs) since two randomized trials show that the use of celecoxib or naproxen could not prevent AD, or improve the cognitive function of AD patients [13,14]. A clinical trial shows that NSAIDs act differently at various stages of the disease and even have an adverse effect at later stages of AD [15]. So, the immediate goal in experimental research is to focus on the specific COX-dependent signaling sub-pathways and thus to observe the potential therapeutic effect for modifying AD development and avoiding the toxicity of NSAIDs.

PGE2, a product of PGH2 catalyzed by PGE synthase (PGES), can activate the G-protein-coupled PGE receptors (EP), including EP1, EP2, EP3 and EP4. Blockage of EP1 signaling can partially protect neurons from cerebral ischemic injury and suppress Aβ accumulation [16,17]. However, activation of EP1 leads to neurodegeneration, which can be prevented by the use of selective EP1 antagonists [18]. PGE2 is able to both stimulate amyloid-β formation and reduce the occurrence of amyloid-β-induced neurodegeneration [19,20]. Activation of EP3 contributes to ischemic excitotoxicity in ischemic stroke models [21]. Administration of an EP4 antagonist can suppress the development of experimental allergic encephalitis (EAE) *in vivo* [22]. Oral administration of AE3-208, a specific EP4 antagonist, will improve the cognitive performance of APP23 mice, transgenic mice expressing mutant APP [23]. However,it was reported that the activation of EP4 has antiinflammatory effects [24].

The formation of PGD2 is induced by PGD synthase (PGDS) on PGH2. PGD2 receptor (DP) has two subtypes viz. DP1 and DP2. The activation of DP1 is primarily associated with anti-inflammation, but DP1 also has proinflammatory effects [25]. Since DP2 is involved in the development of inflammatory diseases, its blockage may be a novel therapeutic way for control of brain damages and neurodegeneration [26].

Prostacyclin (PGI2) is derived from PGH2 via the action of PGI2 synthase (PGIS) and acts mainly on the membrane-bound PGI2 receptor (IP). As reported, IP knockout (IP−/−) mice suffered from more severe myocardial ischemic injury compared with their wild-type counterparts [27]. PGI2 analogs can prevent ischemia reperfusion brain damage in gerbils and hypertensive rats [28]. PGH2 can be converted by Thromboxane A2 synthase (TXS) to TXA2, and then TXA2 activates TXA2 receptor (TP), which plays a pathophysiological role in the development of cardiovascular diseases and stroke. Presynaptic activation of TP will improve the glutamate release, while postsynaptic activation will inhibit synaptic transmission [29]. A selective TP antagonist could prevent atherothrombosis and ischemic stroke [30].

PGF2α is a major prostanoid biosynthesized from PGH2 by PGF synthase (PGFS), and may undertake some important pathophysiological functions via PGF2α receptor (FP) in an autocrine or paracrine manner. The use of FP−/− mice and FP inhibitor indicates that FP could enhance brain damage by cerebral ischemia and excitotoxicity insult [31,32].

These studies indicate the presence of a much complex PG network in the COX downstream signaling pathways and that it is unclear which strategy should be employed for treatment of brain damage and neurodegeneration*:* to activate or block the same PG receptor. The debate in these experimental findings can be attributed to the differences in tissue sources, methodologies, and especially animal models. Therefore, it is necessary to simultaneously observe the changes of PG synthases/PGs content/PG receptors pathways with the same animal model.

The present study was designed to simultaneously observe the contents of PGs (PGE2, PGD2, TXA2, PGI2, and PGF2α), and the expressions of PG synthases (PGES, PGDS, TXAS, PGIS, and PGFS) and PG receptors (EP1-4, DP1-2, FP, IP and TP) in rat hippocampus after chronic administration of aluminium gluconate. The results in this study will help to explore the mechanism of aluminium neurotoxicity and the importance of COX-2 downstream signaling pathways to the occurrence of chronic brain damage.

## Methods

### Reagents

The following reagents were obtained commercially: a BioFlux reverse transcription (RT) kit and a BIOZOL^™^ total RNA extraction kit (Hangzhou Bioer Technology Co., Ltd.); a Premix PCR kit (Beijing ComWin Biotech Co., Ltd.); mouse antirat β-actin monoclonal antibodies and goat antimouse horseradish peroxidase (HRP)-conjugated secondary antibodies (Zhongshan Goldbridge Technology Co., Ltd., Beijing); rabbit antirat FP (DP1, EP1-4, IP and TP) monoclonal antibodies and goat antirabbit HRP-conjugated secondary antibodies (Cayman, USA); a BeyoECL chemiluminescence detection kit and a bicinchoninic acid (BCA) protein detection kit (Beyotime Institute Biotechnology, Shanghai).

### Animals and experimental protocol

A total of 34 male Sprague Dawley rats, aged 8 weeks old and weighing 200–250 g at the beginning of experiments, were purchased from the Laboratory Animal Center of Chongqing Medical University (No. SCXK (Yu) 2010–0001). They were housed in standard conditions: 22 ± 2°C, 50 ± 2% humidity, and 12 h light/dark cycles (light from 8:00–20:00). All experimental procedures on animals were approved by the Chongqing Medical University’s Institutional Animal Ethics Committee. The 34 rats were randomly divided into two groups: a control group and an Al-treated group (each n = 17). The Al-treated group received chronic i.g. administration of aluminium gluconate (Al^3+^ 200 mg/kg per day), 5 days per week for 20 continuous weeks. The control group was treated with an equal volume of sodium gluconate [11,33,34].

### Morris Water Maze test

On the following day after aluminium gluconate administration was completed, the rats in each group were subjected to a spatial learning and memory test using a DMS-2 Morris water maze (Institute of Materia Medica, Chinese Academy of Medical Sciences, Beijing) with a height of 0.5 m, diameter of 1.5 m, water depth of 0.4 m and temperature of 23 ± 2°C. The rats were allowed to learn how to navigate the maze for four days. On the fifth day, spatial memory was tested as previously reported [35]. The rats in each group received a probe trial in which the platform was removed. The time for a rat to pass through the place, where the platform was previously placed, was called the time to explore the platform (exploring time).

### Pathomorphological observation

Six days after aluminium gluconate administration was completed, 4 rats from each group were selected for histopathological observation. According to a reported method [33], the rats were intraperitoneally anesthetized with 1 mL/100 g of 4% chloral hydrate and transcardially perfused with 150 mL of 0.9% saline containing 375U heparin, as well as 200 mL of 4.0% paraformaldehyde fixing solution containing 0.1 M phosphate buffer solution (PBS, pH 7.2). The cerebral tissue was isolated and made into 5-μm-thick coronal sections, which were stained by hematoxylin and eosin (H&E). The pathological changes in hippocampal nerve cells were observed by light microscopy. Injured neurons and dead nerve cells were identified by their acidophilic (eosinophilic) cytoplasm, pyknotic nuclei and nucleoli disappearance under light microscopy [36]. Ten consecutive fields were selected from the dorsal hippocampal CA1 subfield of each section. Then the whole numbers of intact nerve cells were counted using a microscope at 400× and the extent of cell death was estimated.

### Measurement of malondialdehyde (MDA) content and superoxide dismutase (SOD) activity

Six days after aluminium gluconate administration was completed, the brains of four rats from each group were removed and frozen at −80°C. Hippocampal tissues were homogenized with saline at a weight- volume ratio of 1:9, followed by centrifugation. The supernatants were collected to detect MDA content and SOD activity according to the manufacturer’s manual (Jiancheng Bioengineering Ltd, Nanjing). The protein content of each sample was measured by biuret spectrophotometry.

### Detection of PGE2, PGD2, TXA2, PGI2, and PGF2α levels

Six days after aluminium gluconate administration was completed, the brains of the four rats from each group were removed. Hippocampal tissue homogenates from each group (n = 4) were prepared with saline at a weight-volume ratio of 1:9 and then centrifuged at 14000 g × 10 min under 4°C. After that, the supernatants were collected for measurement of PGE2, PGD2, TXA2, PGI2, and PGF2α levels according to the manual of the enzyme-linked immunosorbent assay (ELISA) kit (Cayman, USA). Since PGI_2_ and TXA_2_ can be easily metabolized, their levels were represented by their metabolites 6-keto-PGF_1α_ and TXB_2_, respectively. In order to avoid the post-mortem production of prostanoids from oxylipids, we added indomethacin, a nonselective inhibitor of COX-1 and COX-2, according to the manual of the ELISA kit.

### Reverse transcription polymerase chain reaction (RT-PCR) of mRNA of PG synthases and PG receptors

After the learning and memory function test was completed, brain hippocampi of four rats from each group were removed and frozen at −80°C. Then the mRNA expressions of PG synthases and PG receptors in the hippocampi were detected by RT-PCR. The tested PG synthases include cytosolic PGES (cPGES), microsomal PGES (mPGES)-1, hematopoietic PGDS (H-PGDS), prostacyclin synthase (PGIS) and thromboxane synthase (TXAS). The mRNA expression of lipocalin-type PGDS (L-PGDS) was not measured in this study since its cDNA sequences could be found in NCBI Genbank. The tested PG receptors include EP1-4, DP1, FP, IP and TP. Total RNA was extracted from rat brain hippocampi using BIOZOL reagents according to the directions of the total RNA extraction kit. The primer sequences used for RT-PCR amplification and the lengths of the products are listed in Table 1.

**Table 1:** **The primer sequences for RT-PCR amplification and lengths of the products**

**mRNA primer**	**Sequence**	**Product length**
H-PGD	F: 5’-TCTTGGGTCTCTTGGGATTTC-3’	474 bp
	R: 5’- GGTCCTTGCTAAAGGTGATGA-3’	
cPGES	F: 5’-GGTACGACCGAAGGGACTATG-3’	421 bp
	R: 5’-GAATCATCATCTGCTCCGTCT-3’	
mPGES-1	F: 5’-GTGATGGAGAACAGCCAGGT-3’	307 bp
	R: 5’-TGAGGACCACGAGGAAATGTA-3’	
PGIS	F: 5’-TTTTACAGATGACCGCACTCC-3’	416 bp
	R: 5’-GAAATGAGTCAGCAGCAGGAC-3’	
TXAS	F: 5’-TGACTCTGTCCGTGGTTCTCT-3’	492 bp
	R: 5’-AACACCTCTGGATGTCGAATG-3’	
DP1	F: 5’-TTCTACCAAAGGCACATCACC-3’	390 bp
	R: 5’-AGCCAGCAGAACAAAGTGGT-3’	
EP1	F: 5’-CAGGTGAAGTGGATCTGAAAGG-3’	359 bp
	R: 5’-GACGAACAACAGGAAGGTGGC-3’	
EP2	F: 5’-GCGAGAGTCGTCAGTATCTCCT-3’	400 bp
	R: 5’-CGCCTGTAGAAGTAAGGGTGTC-3’	
EP3	F: 5’-GTATGCCAGCCACATGAAGAC-3’	376 bp
	R: 5’-ACACATGATCCCCATAAGCTG-3’	
EP4	F: 5’-GTGCTCATCTGCTCCATTCCG-3’	380 bp
	R: 5’-CGAGGCTGCTTTCAGTTAGGT-3’	
FP	F: 5’-AATAATTCCCCAGTGACCTGTG-3’	254 bp
	R: 5’-GATGCTTGCTGATTCTCCTTCT-3’	
IP	F: 5’-CCTCTCATTGTAGGTGGCAGA-3’	416 bp
	R: 5’-GCGTACAGGTAGGGATGACTG-3’	
TP	F: 5’-AGATGATGGTTCAGCTCGTAGG-3’	403 bp
	R: 5’-GTAACTCCATCCCACCAAACAT-3’	
β-actin	F: 5′-cacccgcgagtacaaccttc-3′	207 bp
	R: 5′- cccatacccaccatcacacc-3	

Two-step RT-PCR was carried out according to directions of the BioFlux RT kit and the Premix PCR kit. The total reaction volume for RT was 10 μL, containing 2 μL of 5× RT buffer, 1 μL of 10 mM dNTP mixture, 0.5 μL of 10 pmol/μL oligo (dT), 0.5 μL of 10 U/μL RNase inhibitor, 0.5 μL of AMV reverse transcriptase (5 U/μl), 1 μL (1 μg) of RNA template, and 4.5 μL of RNase-free H_2_O. The RT conditions were 50°C for 45 min, 95°C for 5 min and ice-bath for 5 min. The total volume for PCR was 25 μL, containing 12.5 μL of 2 × Taq MasterMix, 1.0 μL of cDNA, 1.0 μL of 10 μM forward primer, 1.0 μL of 10 μM reverse primer, and 9.5 μL of sterile double-distilled water. The PCR conditions were 94.0°C for 2 min, 35 cycles of 94.0°C for 30 s, 57.8°C for H-PGDS (57.4 for cPGES, 59.4 for mPGES-1, 59.7 for PGIS mRNA, 57.4 for TXAS, 57.0 for DP1, 59.0 for EP1, 57.0 for EP2, 57.0 for EP3, 59.4 for EP4, 57.0 for FP, 57.4 for IP, 57.0 for TP and 57.0 for β-actin) for 30 s and 72.0°C for 30 s, followed by extension at 72.0°C for 5 min.

The PCR products were visualized after 2% agarose gel electrophoresis. The integrated gray values of the product bands were measured using a BioRad gel imaging and analysis system (Hercules, CA, USA). The mRNA levels of cPGES, mPGES-1, H-PGDS, PGIS, TXAS, EP1-4, DP1, FP, IP and TP were calculated as ratios to the corresponding β-actin mRNA level.

### Western blot analysis of PG receptor protein expressions

The hippocampi (50 mg) were added with 0.5 mL of a tissue lysate solution (Beyotime) and then the proteins were extracted, followed by centrifugation at 14,000 × *g* and 4°C for 10 min. The supernatant was collected for Western blot analysis. The protein content was detected using the BCA method according to the directions of the protein detection kit (Beyotime).

Then proteins were separated by sodium dodecyl sulfate polyacrylamide gel electropheresis (SDS-PAGE) with 5% stacking gel and 10% separating gel. The proteins were transferred to polyvinylidene difluoride (PVDF) membranes, which were blocked for 1.0 h with PBS containing 5% fat-free milk (weight/volume). The blots were incubated at 4°C overnight with primary antibodies at dilution 1:300 for TP, IP, EP3 and DP1, at 1:400 for EP2, EP4 and mPGES-1, at 1:500 for FP, and at 1:1000 for β-actin. The blots were then incubated at 37°C for 2 h with a secondary antibody at 1:2000 for β-actin, and 1:5000 for PG receptors. The immunoreactive bands of PG receptors and β-actin were visualized with a BeyoECL plus chemiluminescence detection kit, and the optical density bands were detected using a BioRad gel imaging and analysis system. The protein levels of PG receptors were calculated as ratios to the corresponding β-actin protein level.

### Statistical Analysis

All data are expressed as means ± standard deviation (SD). Statistical analysis was carried out with SPSS 17.0 (IBM Corporation, Armonk, NY, USA). Between-group differences in behavior test were evaluated by two-way analysis of variance (ANOVA). Except for the behavior test, between-group differences were evaluated by one-way ANOVA and then Student’s *t*-test. *P* < 0.05 was considered significant.

## Results

### Spatial learning and memory functions of rats

At the stage of spatial learning function test from d 3 to d 4, the time for the Al-treated mice to learn to navigate the maze was significantly prolonged (p < 0.05 and p < 0.01); at the stage of spatial memory function test on d 5, the exploring time was significantly longer (p < 0.01) compared with the control group (Table 2).

**Table 2:** **Changes of spatial learning and memory function of rats caused by aluminium gluconate overload (n = 17)**

	**Exploring time(s)**				
	**Day 1**	**Day 2**	**Day 3**	**Day 4**	**Day 5**
Control group	71.88 ± 12.33	47.55 ± 17.54	22.33 ± 10.12	18.37 ± 8.39	15.03 ± 4.55
Al-treated group	75.84 ± 10.42	52.63 ± 19.49	36.39 ± 19.65^*^	31.67 ± 16.84^**^	29.08 ± 17.56^**^

Data are expressed as mean ± SD of seventeen individual experiments. ∗P < 0.05 compared with corresponding control group; ∗∗P < 0.01 compared with corresponding control group.

### Morphological changes of rat hippocampus

**Figure 1:**
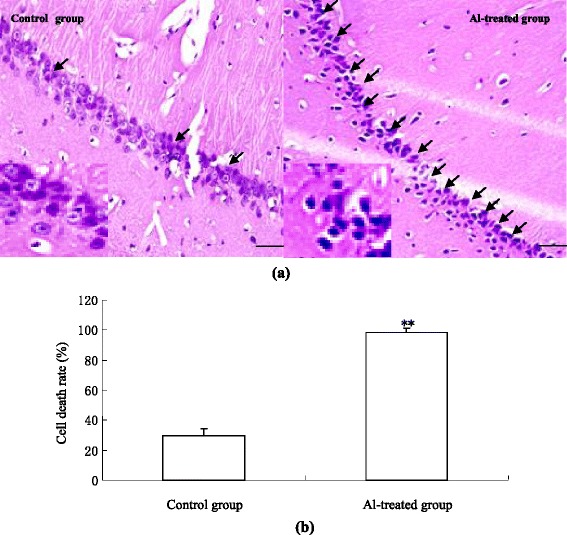
**Morphological changes of rat hippocampal neuron induced by aluminium gluconate overload. (a)** Representative graph of HE-stained CA1 section, 400×. Scale bars = 50 μm. Arrow indicates cell karyopyknosis. Dead nerve cells are characterized by eosinophilic changes; for instance, nerve cells became deep and red with nuclear pyknosis and nucleoli disappearance under light microscopy. **(b)** Data showing the cell death rate (n = 4). Data are expressed as mean ± SD of four individual experiments. ∗P < 0.05 compared with control group.

In the control group, the hippocampal nerve cells were closely arranged and well structured, and the morphological structure kept clear and intact. However, in the Al-treated group, the nerve cells of hippocampal CA1 subfield were found with significant karyopyknosis and loss (Figures 1a and b).

### SOD activity and MDA content in rat hippocampus

As shown in Table 3, hippocampal SOD activity significantly decreased and MDA content significantly increased in the Al-treated group compared with the control group, P < 0.05.

**Table 3:** **Changes of SOD activity and MDA content in rat hippocampus caused by aluminium gluconate overload (n = 4)**

	**SOD (U/mg prot)**	**MDA(nmol/mg prot)**
Control group	40.06 ± 4.31	2.70 ± 1.40
Al-treated group	26.02 ± 7.61^*^	4.64 ± 0.70^*^

Data are expressed as mean ± SD of four individual experiments. ∗P < 0.05 compared with control group.

### Levels of PGE2, PGD2, TXB_2_, 6-keto-PGF_1α_, and PGF2α in rat hippocampi

The levels of 6-keto-PGF1α, PGE2, TXB2, PGF2α and PGD2 in rat hippocampi in the Al-treated group significantly increased by 251.20%, 179.76%, 81.17%, 80.93% and 76.38%, respectively, compared with the control group (Table 4).

**Table 4:** **Changes of PG levels in rat hippocampus caused by aluminium gluconate overload (n = 4, ng/g tissue)**

	**PGD2**	**PGE2**	**PGF2α**	**6-keto-PGF1α**	**TXB2**
Control group	101.47 ± 38.01	42.09 ± 6.92	24.23 ± 9.20	7.91 ± 0.93	18.22 ± 5.86
Al-treated group	178.97 ± 26.44*	117.75 ± 24.51**	43.84 ± 12.74*	27.78 ± 4.90**	33.01 ± 4.62*

Data are expressed as mean ± SD of four individual experiments. ∗P < 0.05 compared with corresponding control group; ∗∗P < 0.01 compared with corresponding control group.

### Expression of PG synthase mRNAs in rat hippocampi

**Figure 2:**
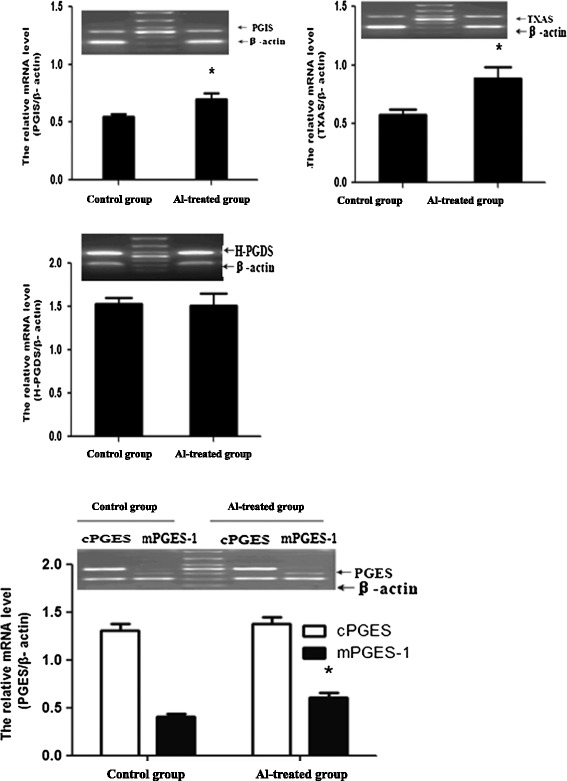
**Expression of PG synthase mRNA in rat hippocampus (n = 4).** The expression of PG synthase mRNA was measured by RT-PCR. The middle band on the PCR images is a marker. The relative mRNA level of PG synthase was standardized to endogenous β-actin mRNA for each sample. Data are expressed as mean ± SD of four individual experiments. Al administration induced a significant increase of TXAS, mPGES-1 and PGIS compared with the control group (∗P < 0.05).

The rat hippocampal mRNA expressions of TXAS, mPGES-1 and PGIS significantly increased by 53.45%, 48.78% and 27.27% respectively in the Al-treated group compared with the control group (Figure 2).

### mRNA and protein expressions of PG receptors in rat hippocampi

**Figure 3:**
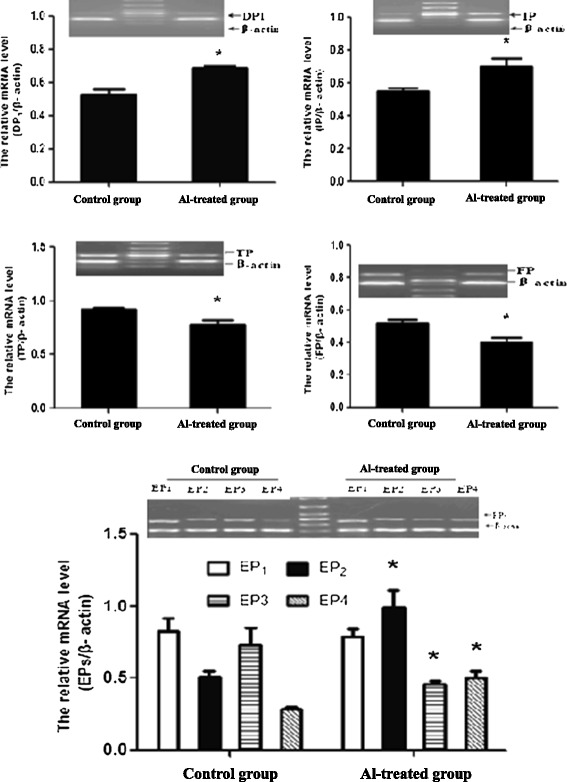
**Expression of PG receptors mRNA in rat hippocampus (n = 4).** The mRNA expression of PG receptors was measured by RT-PCR. The middle band on the PCR images is a marker. The relative mRNA level of PG receptors was standardized to endogenous β-actin mRNA for each sample. Data are expressed as mean ± SD of four individual experiments. Al administration induced a significant increase of EP2, EP4, DP1 and IP levels and a significant decrease of EP3, FP and TP levels compared with the control group (∗P < 0.05).

The rat hippocampal mRNA expressions of EP2, EP4, DP1 and IP significantly increased by 94.12%, 72.41%, 38.00% and 27.27%, respectively, while the mRNA expressions of EP3, FP and TP significantly decreased by 36.99%, 15.38% and 15.22%, respectively in the Al-treated group compared with the control group (Figure 3).

**Figure 4:**
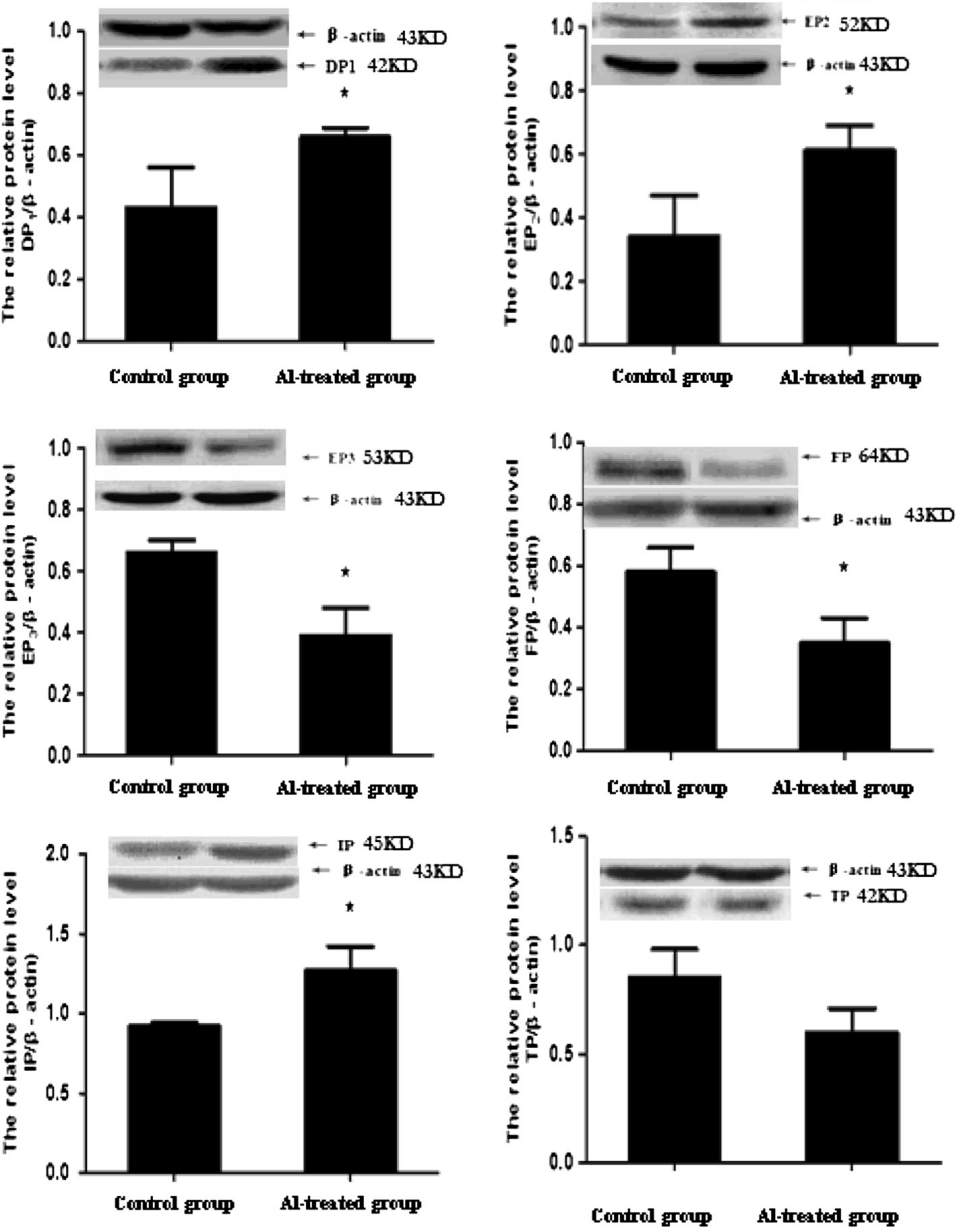
**Protein expression of PG receptors in rat hippocampus (n = 4).** The protein expression of PG receptors was measured by Western blot. The relative protein level of PG receptors was standardized to endogenous β-actin protein for each sample. Data are expressed as mean ± SD of four individual experiments. Al administration induced a significant increase of EP2, DP1 and IP levels and a significant decrease of EP3 and FP levels compared with the control group (∗P < 0.05).

The rat hippocampal protein expressions of EP2, DP1 and IP increased significantly by 79.41%, 53.49% and 38.04%, respectively, while the protein expressions of EP3 and FP significantly decreased by 40.90% and 39.66%, respectively in the Al-treated group (Figure 4).

## Discussion

Any morphological alteration of hippocampal neurons will result in cognitive impairment, which is the most common hallmark for brain damages and neurodegeneration [37]. Therefore, in this study, we observed the changes of neuroinflammation, oxidative stress and neuronal loss in chronic aluminium gluconate-overloaded rat hippocampi. The experimental results show that chronic aluminium gluconate administration not only caused significant hippocampal neuron damage and oxidative stress response in rats, but also resulted in the impairment of spatial learning and memory functions. These results are similar with our previous study [11].

Aluminum overload caused a significant reduction of SOD activity and a significant increase of MDA content. Our previous study shows that chronic aluminium gluconate exposure caused significant increase of Al, Fe, Mn, Cu and Zn levels in rat hippocampi [11]. The brain accumulation of Al, Fe, Mn, Cu and Zn could induce a significant oxidative stress, which will cause tissue damage and AA formation by degradation of cell membrane phospholipids.

our results show that PGD2 level is the highest in normal rat hippocampus, followed by PGE2, PGF2α, TXB2 and 6-keto-PGF1α. Compared with the control group, the treatment of aluminium gluconate significantly increased rat hippocampal 6-keto-PGF1α, PGE2, TXB2, PGF2α and PGD2 levels by 251.20%, 179.76%, 81.17%, 80.93% and 76.38%, respectively. Similarly, PGD2 was also the most abundant prostanoid in rat brain tested by gas-chromatography-mass spectrometry (GC-MS) [38]. PGD synthase (PGDS) has two isoenzymes of L-PGDS and H-PGDS. L-PGDS is localized in oligodendrocyte and neurons [39], and the inhibition of its activation and expression could abate neuronal injury [40,41]. However, L-PGDS is not the major part responsible for PGD2 formation and does not influence the effects of PGD2 [42]. H-PGDS is abundantly expressed in the microglia of cerebellum and the hippocampal neurons of CNS [43]. PGD2 functions by activating two G-protein-coupled receptors viz. DP1 and DP2. DP1 and DP2 have opposite effects on neurons subjected to injury factors. As reported, physiological concentrations of PGD2 and a selective DP1 agonist BW245C protect the hippocampal neurons from glutamate toxicity. Meanwhile, the activation of DP2 will cause significant neuron loss [43]. DP1(−/−) mice are in a larger infarct size than wild-type mice subjected to middle cerebral artery occlusion (MCAO)-induced brain damage [44]. Young and aged DP1-deficient mice are more susceptible to excitotoxicity than controls, and DP1 agonist will attenuate both acute and chronic brain damages [45]. Our experimental data show that PGD2 content and DP1 mRNA/protein expressions in rat hippocampal neurons significantly increased in the Al-treated group compared with the control group. However, the importance of the change of PGD2/DP1 signaling pathway is unclear in rats with brain damage caused by chronic aluminium gluconate overload. Combined with previous studies, we think that the elevation of PGD2 level resulted from the increase of COX expression and PGH2 level. Moreover, aluminium gluconate administration caused a protective increase of DP1 expression. These results indicate that the PGD2/DP1 signaling pathway is protective against brain damage induced by chronic aluminium gluconate overload. However, this hypothesis needs to be confirmed by a number of experiments.

PGE synthases (PGES) consist of three major isozymes: microsomal PGES-1 (mPGES-1), mPGES-2 and cytosolic PGES (cPGES). mPGES-1 is inducible, while mPGES-2 and cPGES are constitutive. PGE2 as the product of PGES exerts complex effects through four PGE receptors: EP1, EP2, EP3, and EP4. Our results show that PGE2 content in rat hippocampus significantly increased in the Al-treated group vs. the control group. Administration of aluminium gluconate resulted in significant increase of mPGES-1, EP2 and EP4 mRNA expressions and EP2 protein expression, and also caused significant decrease of EP3 mRNA and protein expressions. It was similarly reported that protein and mRNA expressions of COX-2 and mPGES-1 significantly increased in postmortem brains of AD patients [10]. Activation of EP1 which is expressed widely in brain neurons would reduce the Aβ neurotoxicity to murine primary cortical neurons and a human neuroblastoma cell line [46]. Conversely, EP1 is the neurotoxic effector of downstream COX-2, and the blockage of EP1 signaling protects against cerebral ischemic damage and Aβ neurotoxicity [16,17]. EP2 signaling is very important for synaptic plasticity, and its selective allosteric potentiator has a neuroprotective effect [47,48]. However, activation of PGE2 could stimulate the production of Aβ by coordination with EP4 [21]. The cerebral infarct volume decreases in EP3 (−/−) mice [49]. Selective activation of EP3 can dose-dependently increase the infarct volume in MCAO mice [50]. However, another study shows that the infarct volume in EP3 (−/−) mice does not obviously change [51]. Oral administration of a specific EP4 antagonist caused an improvement of learning and memory functions and a decrease of brain Aβ level in APP23 mice [23]. Obviously, knockout of EP4 and administration of an EP4 antagonist can both suppress the development of encephalomyelitis [22]. However, it is still unclear whether the decrease of EP3 expression and the increase of EP2 and EP4 expressions are beneficial or harmful to treatment of chronic brain damage. Therefore, more experiments with agonists and antagonists of EP2, EP3 and EP4 should be carried out to explore the therapeutic values from the changes of EPs.

PGF2α is a major PG biosynthesized by PGFS from PGH2. An immunohistological study shows that PGF2α production in hippocampal neurons can be immediately induced by injection of kainic acid, and that PGF2α-immunopositive neurons also express COX-2 and FP [52]. Studies on FP blockade and FP knockout *in vivo* and *in vitro* suggest that FP contributes to the prevention of ischemic and excitotoxic brain damages [31,32]. In our study, however, chronic aluminium gluconate administration caused a significant increase of PGF2α level and obviously downregulated the mRNA and protein expressions of FP in rat hippocampus. It is still unclear whether the activation of FP signaling pathway is beneficial or harmful for acute and chronic brain damages.

PGI2 or prostacyclin, which is derived from AA via PGIS, exerts its important physiological functions by acting mainly on IP. Besides endothelial cells, PGIS is also abundantly expressed in hippocampus and cortex neurons companied with the expression of IP. Cerebral ischemia reperfusion insult will cause a delayed and transient induction of PGIS in hippocampus and cortex neurons [30]. COX-1/PGIS adenoviral gene transfer to substantia nigra will prevent dopamine depletion and behavioral deficits induced by 6-OHDA in rats [53]. The infarct volume and behavioral damage in IP−/−mice were significantly enhanced compared with the wild-type mice, and administration of beraprost, a selective IP agonist, significantly improved the neurological deficit and decreased the infarct volume in wild-type mice [54]. Our experiments also show that 6-keto-PGF_1α_ content and the expressions of IP mRNA and protein obviously increased in the hippocampi of the Al-treated rats. These results suggest that activation of PGIS/IP signaling pathway will prevent brain damage.

Evidence shows that postsynaptic TP activation will inhibit synaptic transmission while presynaptic activation will promote the glutamate release, which mediates the excitotoxic damage to the brain [29]. Administration of a selective TP antagonist could prevent ischemic stroke [54]. The present study shows that the TXAS mRNA expression and TXB2 content in the Al-treated rat hippocampi increased significantly. The hippocampal mRNA levels of TP in the Al-treated rats significantly decreased but the protein levels were unchanged. These results suggest that the intervention of the TXAS/TP signaling pathway has no important value for treatment of chronic brain damage induced by aluminium gluconate overload.

In conclusion, our study confirms the disturbance of a PGS/PGs/PG receptors signaling pathway in rat hippocampi caused by chronic aluminium gluconate overload and this may be involved in the mechanism of aluminium neurotoxicity. However, the value of such changes for this signaling pathway is not completely clear. We are starting a series of studies with agonists and antagonists of DP1-2, IP, FP, TP and EP1-4, and aim to explore the therapeutic value of these changes of PGS/PGs/PGs receptors signaling pathways for treatment of acute and chronic brain damages in rats.

## Abbreviations

Al: Aluminium
AD: Alzheimer’s disease
IL: Interleukin
TNF: Tumor necrosis factor
AA: Arachidonic acid
PLA2: Phosphalipase A2
PG: Prostaglandin
COX: Cyclooxygenase
TXA2: Thromboxane A2
NSAIDs: Non-steroidal antiinflammatory drugs
PGES: PGE synthase
Aβ: Amyloid beta
APP: Amyloid beta precursor protein
PGFS: PGF synthase
CNS: Central nervous system
BCA: Bicinchoninic acid
H&E: Hematoxylin and eosin staining
MDA: Malondialdehyde
SOD: superoxide dismutase
ELISA: Enzyme-linked immunosorbent assay
RT-PCR: Reverse transcription polymerase chain reaction
H-PGDS: Hematopoietic PGD synthase
PVDF: Polyvinylidene difluoride
SD: Standard deviation
ANOVA: Analysis of variance
L-PGDS: Lipocalin-type PGD synthase
mPGES-1: Microsomal PGES-1
cPGES: Cytosolic PGES
PGFS: PGF synthase
PGD: Receptor
DP: PGE receptor
EP: PGF receptor
FP: Thromboxane A2 receptor
TP: PGI2 synthase
PGIS: Prostacyclin receptor: IP

## References

[1] Shaw CA, Tomljenovic L (2013). Aluminium in the central nervous system (CNS): toxicity in humans and animals, vaccine adjuvants and autoimmunity. Immunol Res.

[2] Wang J, Hihara E (2004). A unified formula for calculating body surface area of humans and animals. Eur J Appl Physiol.

[3] Frisardi V, Solfrizzi V, Capurso C, Kehoe PG, Imbimbo BP, Santamato A (2010). Aluminium in the diet and alzheimer’s disease: from current epidemiology to possible disease-modifying treatment. J Alzheimer’s Dis.

[4] Campbell A (2006). The role of aluminium and copper on neuroinflammation and Alzheimer's disease. J Alzheimers Dis.

[5] Castorina A, Tiralongo A, Giunta S, Carnazza ML, Scapagnini G, D'Agata V (2010). Early effects of aluminum chloride on beta-secretase mRNA expression in a neuronal model of beta-amyloid toxicity. Cell Biol Toxicol.

[6] Giunta S, Andriolo V, Castorina A (2014). Dual blockade of the A1 and A2A adenosine receptor prevents amyloid beta toxicity in neuroblastoma cells exposed to aluminum chloride. Int J Biochem Cell Biol.

[7] Pasinetti GM, Aisen PS (1998). Cyclooxygenase-2 expression is increased in frontal cortex of Alzheimer’s disease brain. Neuroscience.

[8] Manev H, Uz T, Qu T (2000). 5-lipoxygenase and cyclooxygenase mRNA expression in rat hippocampus: early response to glutamate receptoractivation by kainite. Exp Gerontol.

[9] Jalil CE, Falcon GA, Cabrera GM, Alvarez D, Dalain AS, Martinez G (2003). Assessment of the relative contribution of COX-1 and COX-2 isoforms to ischemia-induced oxidative damage and neurodegeneration following transient global cerebral ischemia. J Neurochem.

[10] Rao JS, Rapoport SI, Kim HW (2011). Altered neuroinflammatory, arachidonic acid cascade and synaptic markers in postmortem Alzheimer’s disease brain. Transl Psychiatry.

[11] Yu L, Jiang R, Su Q, Yu H, Yang J (2014). Hippocampal neuronal metal ion imbalance related oxidative stress in a rat model of chronic aluminium exposure and neuroprotection of meloxicam. Behav Brain Funct.

[12] Su Q, Yang J, Zhang P (2009). Effect of meloxicam on cyclooxygenase 2 expression of chronic aluminium overload-induced nerve degeneration in rat hippocampus. Chin J Pharmacol Toxicol.

[13] Lyketsos CG, Breitner JC, Green RC, Martin BK, Meinert C, ADAPT Research Group (2007). Naproxen and celecoxib do not prevent AD in early results from a randomized controlled trial. Neurology.

[14] Martin BK, Szekely C, Brandt J, Piantadosi S, Breitner JC, ADAPT Research Group (2008). Cognitive function over time in the Alzheimer's Disease Anti-inflammatory Prevention Trial (ADAPT): results of a randomized, controlled trial of naproxen and celecoxib. Arch Neurol.

[15] Breitner JC, Baker LD, Montine TJ, Meinert CL, Lyketsos CG, Ashe KH (2011). Extended results of the Alzheimer's disease anti-inflammatory prevention trial. Alzheimers Dement.

[16] Zhen G, Kim YT, Li RC, Yocum J, Kapoor N, Langer J (2012). PGE(2) EP1 receptor exacerbated neurotoxicity in a mouse model of cerebral ischemia and Alzheimer's disease. Neurobiol Aging.

[17] Kawano T, Anrather J, Zhou P, Park L, Wang G, Frys KA (2006). Prostaglandin E2 EP1 receptors: downstream effectors of COX-2 neurotoxicity. Nat Med.

[18] Abe T, Kunz A, Shimamura M, Zhou P, Anrather J, Iadecola C (2009). The neuroprotective effect of prostaglandin E2 EP1 receptor inhibition has a wide therapeutic window, is sustained in time and is not sexually dimorphic. J Cereb Blood Flow Metab.

[19] Hoshino T, Namba T, Takehara M, Nakaya T, Sugimoto Y, Araki W (2009). Prostaglandin E2 stimulates the production of amyloid-beta peptides through internalization of the EP4 receptor. J Biol Chem.

[20] Shie FS, Montine KS, Breyer RM, Montine TJ (2005). Microglial EP2 as a new target to increase amyloid beta phagocytosis and decrease amyloid beta-induced damage to neurons. Brain Pathol.

[21] Ikeda-Matsuo Y, Tanji H, Ota A, Hirayama Y, Uematsu S, Akira S (2010). Microsomal prostaglandin E synthase-1 contributes to ischaemic excitotoxicity through prostaglandin E2 EP3 receptors. Br J Pharmacol.

[22] Yao C, Sakata D, Esaki Y, Li Y, Matsuoka T, Kuroiwa K (2009). Prostaglandin E2-EP4 signaling promotes immune inflammation through Th1 cell differentiation and Th17 cell expansion. Nat Med.

[23] Hoshino T, Namba T, Takehara M, Murao N, Matsushima T, Sugimoto Y (2012). Improvement of cognitive function in Alzheimer's disease model mice by genetic and pharmacological inhibition of the EP(4) receptor. J Neurochem.

[24] Tang EH, Libby P, Vanhoutte PM, Xu A (2012). Antiinflammation therapy by activation of prostaglandin EP4 receptor in cardiovascular and other inflammatory diseases. J Cardiovasc Pharmacol.

[25] Mohan S, Ahmad AS, Glushakov AV, Chambers C, Doré S (2012). Putative role of prostaglandin receptor in intracerebral hemorrhage. Frontiers in Neurol.

[26] Spik I, Brenuchon C, Angeli V, Staumont D, Fleury S, Capron M (2005). Activation of the prostaglandin D2 receptor DP2/CRTH2 increases allergic inflammation in mouse. J Immunol.

[27] Birnbaum Y, Ye Y, Rosanio S, Tavackoli S, Hu ZY, Schwarz ER (2005). Prostaglandins mediate the cardioprotective effects of atorvastatin against ischemia-reperfusion injury beneficial effects of a new prostacyclin analogue, KP-10614, on acute myocardial infarction in rats. Cardiovasc Res.

[28] Matsuda S, Wen TC, Karasawa Y, Araki H, Otsuka H, Ishihara K (1997). Protective effect of a prostaglandin I2 analog, TEI-7165, on ischemic neuronal damage in gerbils. Brain Res.

[29] Hsu KS, Kan WM (1996). Thromboxane A2 agonist modulation of excitatory synaptic transmission in the rat hippocampal slice. Br J Pharmacol.

[30] Chamorro A (2009). TP receptor antagonism: a new concept in atherothrombosis and stroke prevention. Cerebrovasc Dis.

[31] Saleem S, Ahmad AS, Maruyama T, Narumiya S, Doré S (2009). PGF(2alpha) FP receptor contributes to brain damage following transient focal brain ischemia. Neurotox Res.

[32] Kim YT, Moon SK, Maruyama T, Narumiya S, Doré S (2012). Prostaglandin FP receptor inhibitor reduces ischemic brain damage and neurotoxicity. Neurobiol Dis.

[33] Zhang J, Yang JQ, He BC, Zhou QX, Yu HR, Tang Y (2009). Berberine and total base from rhizoma coptis chinensis attenuate brain injury in an aluminium-induced rat model of neurodegenerative disease. Saudi Med J.

[34] Pan Y, Zhang P, Yang J, Su Q (2010). 5-lipoxygenase expression in a brain damage model induced by chronic oral administration of aluminium. Neural Regen Res.

[35] Wang H, Jiang R, He Q, Zhang Y, Zhang Y, Li Y (2012). Expression Pattern of Peroxisome Proliferator-Activated Receptors in Rat Hippocampus following Cerebral Ischemia and Reperfusion Injury. PPAR Res.

[36] Sankar R, Shin DH, Liu H, Mazarati A, Pereira de Vasconcelos A, Wasterlain CG (1998). Patterns of status epilepticus-induced neuronal injury during development and long-term consequences. J Neurosci.

[37] Budson AE (2009). Understanding memory dysfunction. Neurologist.

[38] Abdel-Halim MS, Hamberg M, Sjoquist B, Anggård E (1977). Identification of prostaglandin D_2_ as a major prostaglandin in homogenates of rat brain[J]. Prostaglandins.

[39] Chen CP, Chen RL, Preston JE (2009). Age-related increase of prostaglandin D(2) synthase concentration and glycation in ovine cerebrospinal fluid. Exp Gerontol.

[40] Saleem S, Shah ZA, Urade Y, Doré S (2009). Lipocalin-prostaglandin D synthase is a critical beneficial factor in transient and permanent focal cerebral ischemia. Neuroscience.

[41] Taniguchi H, Mohri I, Okabe-Arahori H, Kanekiyo T, Kagitani-Shimono K, Wada K (2007). Early induction of neuronal lipocalin-type prostaglandin D synthase after hypoxic-ischemic injury in developing brains. Neurosci Lett.

[42] Kanekiyo T, Ban T, Aritake K, Huang ZL, Qu WM, Okazaki I (2007). Lipocalin-type prostaglandin D synthase/beta-trace is a major amyloid beta-chaperone in human cerebrospinal fluid[J]. Proc Natl Acad Sci U S A.

[43] Suk K (2012). Unexpected role of lipocalin-type prostaglandin D synthase in brain. Cell Adhesion Migration.

[44] Saleem S, Zhuang H, de Brum-Fernandes AJ, Maruyama T, Narumiya S, Doré S (2007). PGD2 DP1 receptor protects brain from ischemia–reperfusion injury. Eur J Neurosci.

[45] Ahmad AS, Ahmad M, Maruyama T, Narumiya S, Doré S (2010). Prostaglandin D2 DP1 receptor is beneficial in ischemic stroke and in acute exicitotoxicity in young and old mice. Age.

[46] Li X, Rose SE, Montine KS, Keene CD, Montine TJ (2013). Antagonism of Neuronal Prostaglandin E2 Receptor Subtype1 Mitigates Amyloid β Neurotoxicity *In Vitro*. J Neuroimmune Pharmacol.

[47] Yang H, Zhang J, Breyer RM, Chen C (2009). Altered hippocampal long-term synaptic plasticity in mice deficient in the PGE2 EP2receptor. J Neurochem.

[48] Jiang J, Ganesh T, Du Y, Thepchatri P, Rojas A, Lewis I (2010). Neuroprotection by selective allosteric potentiators of the EP2 prostaglandin receptor. Proc Natl Acad Sci U S A.

[49] Ahmad M, Ahmad AS, Zhuang H, Maruyama T, Narumiya S, Doré S (2007). Stimulation of prostaglandin E2–EP3 receptors exacerbates stroke and excitotoxic injury. J Neuroimmunol.

[50] Saleem S, Kim YT, Maruyama T, Narumiya S, Doré S (2009). Reduced acute brain injury in PGE2 EP3 receptor-deficient mice after cerebral ischemia. J Neuroimmunol.

[51] Li J, Liang X, Wang Q, Breyer RM, McCullough L, Andreasson K (2008). Misoprostol, an anti-ulcer agent and PGE2 receptor agonist, protects against cerebral ischemia. Neurosci Lett.

[52] Takei S, Hasegawa-Ishii S, Uekawa A, Chiba Y, Umegaki H, Hosokawa M (2012). Immunohistochemical demonstration of increased prostaglandin F2α levels in the rat hippocampus following kainic acid-induced seizures. Neuroscience.

[53] Tsai M, Weng C, Yu N, Liou D, Kuo F, Huang M (2013). Enhanced prostacyclin synthesis by adenoviral gene transfer reduced glial activation and ameliorated dopaminergic dysfunction in hemiparkinsonian rats. Oxid Med Cell Longev.

[54] Saleem S, Shah ZA, Maruyama T, Narumiya S, Dore S (2010). Neuroprotective properties of prostaglandin I2 IP receptor in focal cerebral ischemia. Neuroscience.

